# Social mobility in transition. Value repertoires in higher education and moral meanings of the profession among first-generation graduates in Chile

**DOI:** 10.3389/fsoc.2026.1771893

**Published:** 2026-04-28

**Authors:** Ismael Tabilo-Prieto

**Affiliations:** 1 Universidad Finis Terrae, Faculty of Education and Social Sciences, Santiago, Chile; 2 Universidad Alberto Hurtado, Faculty of Social Sciences, Santiago, Chile

**Keywords:** first-generation graduates, higher education, moral meanings, social mobility, sociology of valuation and evaluation

## Abstract

**Introduction:**

This study examines how first-in-family graduates incorporate value repertoires during university and attribute moral meanings to their professional activity while transitioning from higher education to employment. Its purpose is to analyse the moral dimension of social mobility, focusing on the valuative frameworks through which graduates interpret their educational and occupational experiences.

**Methods:**

Qualitative fieldwork was conducted between July 2024 and November 2025. Twenty semi-structured interviews were carried out with men and women aged 24–30 who had recently graduated from high- and low-mobility degree programs in public and private universities in Santiago, Chile. Participants were selected through purposive sampling, ensuring they were the first professionals in their families, and the data were analyzed using thematic analysis with Atlas.Ti.

**Results:**

The findings reveal that students’ initial experiences at university are marked by insecurity and a sense of inadequacy. Over time, they begin to value diversity, alongside personal effort and aesthetic taste, transforming the initial shock into diversification of valuative criteria. The appreciation of diversity is, however, coupled with a critique of economic injustice and the development of a sense of inequality. Conceptions of professional life reveal that the university degree is both a credential and a moral symbol, as it embodies dignity and respect. The moral meanings attributed to the profession reflect an interaction between values socialized within the family, mainly vocational and material, and those socialized during university and early professional experiences, such as social contribution.

**Discussion:**

Social mobility through higher education involves moral transformations and redefinitions of self-worth. For first-in-family graduates, university enables new valuative repertoires, expanded horizons, and the possibility of reinterpreting one’s social position. The study contributes to the sociology of valuation and evaluation by showing how moral categories are mobilized in everyday practices of meaning-making. These categories are not abstract but situated, emerging from the lived experiences of students negotiating recognition and self-worth in stratified educational institutions. This research invites further reflection on how educational systems shape not just careers but moral worlds and social imaginaries of possible futures.

## 1 Introduction

Chile has experienced a notable increase in higher education coverage over the last 40 years. This growth and diversification of higher education institutions respond, on the one hand, to the increasing demand of the Chilean population for tertiary education and, on the other hand, to the significant increase in private provision driven by the reform of the 1980s during the military dictatorship (Brunner, 2015). The expansion in enrolment, together with equity-oriented programs to access higher education implemented in subsequent decades, has enabled the emergence of new student profiles, increasing the socioeconomic diversity of the system as a whole (Orellana, 2011; Chiroleu, 2013). Working-class students and those who will become the first professionals in their families today represent what has been described as “new middle groups” (Torche and Wormald, 2004; Espinoza et al., 2013).

The ways in which the middle class has been classified have been widely studied (Barozet and Espinoza, 2008; Espinoza and Barozet, 2009). Within the framework of the so-called neoliberal transformation (Espinoza et al., 2013), these changes include the diversification of market positions resulting from Chile’s economic opening, increased access to credit and consumption, greater participation of women, and delayed entry of young people in the labor market. Together, these changes have enabled new class identities and led to greater horizontal differentiation.

In this context, the massification of higher education is undoubtedly one of the most significant structural changes, particularly in terms of its impact on the middle and working classes. As new student profiles gain access to university through different forms of debt, and since 2016 through the free tuition law, this expansion has significantly affected the educational and occupational aspirations of young people (Sepúlveda and Valdebenito, 2014a). Evidence suggests that these new fractions of the middle-class view access to higher education as a pathway for social mobility and improved quality of life. Particularly in the case of first-generation students, research suggests that they carry a family mandate to compensate for and surpass their parents in terms of education and work, transforming themselves into a kind of middle-class “elite” (Canales et al., 2020).

Although this expansion has made possible new biographical projects, both individual and family-based, the stratified structure of the tertiary level has produced new inequalities (Sepúlveda and Valdebenito, 2014b), primarily reflected in intraclass differentiation and a widespread sense of discontent stemming from the unfulfilled promise of social mobility (Barozet et al., 2021).

Drawing on semi-structured qualitative interviews with first-in-family graduates in Santiago, Chile, this study examines the creation of new value repertoires at university and the moral meanings they attribute to the profession as they transition into employment. Particular attention is given to the valuative frameworks through which lower-middle and working-class graduates evaluate their university experiences and professional activities.

To this end, I draw on the conceptual framework of the sociology of valuation and evaluation (SVE), which has revitalized sociological inquiry into disputed valuation hierarchies that shape processes of classification and the legitimation of worth and recognition (Lamont, 2012; De Munck and Zimmermann, 2014; Cefaï et al., 2015).

The article begins with a brief review of transformations in the tertiary education system in Chile. It then reviews existing evidence on the university experiences of first-generation students. Following this contextual background, the conceptual framework and methodological design of the research are presented. The results are organized into three sections: the experience of inequality and value formation at university, conflicting values about social mobility and moral meaning of professional activity. Finally, the discussion relates the results to the literature and concludes with a summary of the implications of the findings and possible new questions to be addressed.

### 1.1 Promises and paradoxes of higher education expansion in Chile

Over the past four decades, higher education in both Latin America and Chile has undergone a sustained process of expansion and de-elitization, marked by increased market participation and institutional diversification (Baisotti and Pineda-Alfonso, 2022; Labraña and Brunner, 2022). In Chile, this phenomenon became more pronounced in the mid-1980s, with the policies introduced under the dictatorship of Augusto Pinochet. These reforms fragmented the system according to prestige and divided it into three types of institutions: vocational training centers, professional institutes, and universities. This resulted in marked heterogenization, reflected in differences in teaching quality, academic production, institutional capacities, and human resources. At the same time, there was a deliberate effort to reduce the role of the state in university education and to enable the entry of private actors (Bernasconi, 2015). During the 1990s, this trend became more firmly established, though it was accompanied by a significant increase in regulatory and supervisory mechanisms concerning both quality and provision (Santelices V. et al., 2019).

The expansion of enrollment in tertiary education in Chile has been characterized by a cascading pattern, closely associated with the notion of *effectively maintained inequality* (EMI) advanced by Lucas (2001). This means that access is first expanded to high-income groups and then, once their participation is consolidated, it expands downward through processes of institutional stratification. In parallel, university selectivity has operated as the principal mechanism for ordering institutions according to prestige and quality, thereby reproducing stratified access in both academic and social terms (Salazar and Leihy, 2013; Brunner, 2015).

Both the swift proliferation of higher education institutions in the 1980s and their subsequent consolidation in the 1990s were strongly influenced by funding programs such as the Fondo de Desarrollo Institucional (Institutional Development Fund) and the Programa de Mejoramiento de la Calidad y Equidad de la Educación (MECESUP, Program for the Improvement of Quality and Equity in Education), which were designed to raise the quality standards of educational provision. Likewise, in response to persistent inequalities in access, funding mechanisms and admission routes have been diversified in order to expand access opportunities for low-income students (Santelices et al., 2018; Santelices M. et al., 2019; Horn et al., 2019).

Over the last three decades, enrollment growth has been shaped by policies such as teaching scholarships, Bicentennial scholarships, and scholarships for children of education professionals, together with state-guaranteed loans (CAE) and, more recently, the free tuition law. These milestones have helped to expand coverage, although in line with the logic of cascading stratification described above. Indeed, much of this growth has been concentrated in vocational colleges and universities with low selectivity, with a predominance of private providers and a strong preponderance of market criteria in the functioning of the system as a whole (Fleet et al., 2020). This scenario poses new challenges in terms of equity, linked both to the biographical impact of student debt (Pérez-Roa and Gómez Contreras, 2019; Pérez-Roa and Ayala, 2019) and to heterogeneity in the quality of institutions within a highly segmented and diverse system (Chiroleu and Marquina, 2017; Espinoza et al., 2025).

The expansion of enrollment has been also linked to the consolidation of new middle sectors (Torche and Wormald, 2004; Espinoza et al., 2013). Massification has enabled the incorporation of new students from social sectors previously excluded due to their socioeconomic status (Brunner, 2015; Orellana, 2011). Likewise, several changes have taken place in the aspirations and expectations of these groups. Middle-class and working-class families and students have come to see education as an opportunity for social mobility, access to other social groups, increased consumption, and greater well-being in their lives (Pérez-Roa, 2014).

In Chile, evidence suggests that the de-elitization of higher education was based on a promise to improve people’s quality of life through access to better positions in society (Quaresma et al., 2022). Massification has effectively led to increased aspirations among secondary school students to enter tertiary education (Sepúlveda and Valdebenito, 2014b; Duarte and Sandoval, 2018). Accordingly, higher education is considered the best way to combine professional development and personal interests (Valdebenito, 2020).

Nevertheless, a number of studies have shown that higher education may not translate into high rates of return for all graduates (Améstica et al., 2014; Fiscalía Nacional Económica, 2025). Disaggregated analyses by type of institution show varying rates of return for graduates of the same degree program (Urzúa, 2012). Moreover, more recent work has revealed the concentration of economically and culturally elite students in a small group of institutions (Zimmerman, 2019; Villalobos et al., 2020). For graduates carrying student debt, the problem becomes more acute, since they enter the labor market with a substantial financial liability in a context marked by instability and low remuneration (Pérez-Roa and Ayala, 2019).

In relation to the return and overall performance of tertiary education, tensions and trade-offs arise with respect to aspirations and expectations (Vincent-Lancrin, 2017). The expansion of university education and the contraction of formal employment opportunities have produced a vertical “stretching” of stratification (Marginson, 2016). Moreover, research by Campante and Chor (2014) and Brannen et al. (2020) shows that unmet occupational expectations have impacts on political ideologies and stances regarding social discontent. In Chile, young people whose job expectations are not met after graduation tend to develop political ideologies that are more oriented towards supporting pro-equality initiatives (Cox, 2024). Others argue that “degree inflation” and the diminished value of diplomas in the Chilean labor market are key factors behind the widespread discontent among young adults who took part in the 2019 social uprising (Ortúzar, 2024).

The gap between aspirations and professional achievements in the era of massification has been problematized insofar as it produces “unrealistic” optimism (Frye, 2012). Salgado (2024) explores this phenomenon in depth among Chilean secondary school students and concludes that there is a mismatch between aspirations and the capacities to achieve them. This optimism about the future and the high level of ambition appear misaligned when considering the socio-economic status of families.

International evidence shows that this mismatch is a global phenomenon, mainly associated with the role that universities play in modern social imaginaries, which clashes with stratified democratization and the emergence of new forms of inequality (Waller et al., 2017). Higher education systems have been shown to have significant power over the aspirations and expectations of individuals, especially in mass or universal systems. Families and youth often view higher education as a resource for positioning themselves socially and as a means of achieving a universal goal of life improvement (Marginson, 2016). Accordingly, the demand for greater access and the dynamism of these systems have also been driven by families’ ambitions to achieve social status and students’ desire for self-fulfillment (Trow, 2010).

### 1.2 Gains, tears and burdens. The university experience of first-generation students

A high proportion of so-called new students in higher education (Orellana, 2011) are first-generation students, meaning that neither of their parents or guardians completed higher technical or professional studies. The results of the Academic Experience Characterization Questionnaire administered by the Department of Evaluation, Measurement, and Educational Registration in Chile indicate that 43% of students enrolled in higher education in 2024 were first-generation (DEMRE, 2025).

From a sociological perspective, this type of student has been studied as a proxy for social class, especially for the middle and working classes, and as a unit of analysis for social mobility processes at the individual and family level (Beattie, 2018). In the case of Chile, this profile has become the “typical case” of massification and the main representative of intergenerational social mobility. It is estimated that first-generation students come from families in the three lowest income quintiles (Jarpa-Arriagada and Rodríguez-Garcés, 2017) and correspond to a mainly urban profile (Ghiardo and Dávila, 2020).

During their time at university, they have highly complex experiences, marked by difficult academic adaptation, limited social networking, and low participation in extracurricular activities (Castillo and Cabezas, 2010; Flanagan, 2017). Furthermore, in many cases, they must combine work and study, which makes them a “population at risk” given the greater likelihood of dropping out. For them, the probability of obtaining a diploma is 38%, while for their peers with professional parents it rises to 70% (Urzúa, 2012). From a qualitative perspective, Soto (2015) shows that the university experience is strongly linked to parental support. For them, university means at least three things: an opportunity for social mobility, the chance to find their vocation, and a way to repay their parents for their sacrifices.

With regard to their entry into the labor market, Ghiardo and Dávila (2020) highlight that the employment rates of first-in-family graduates reach the same level as those from professional families (more than 90% for men and close to 90% for women). However, they tend to enter low- and middle-income occupations. International evidence suggests that these students have greater difficulty in developing and mobilizing their social and cultural capital (Lehmann, 2019), and face challenging processes when converting the skills acquired at university into capital that is recognized and valued in the workplace (Ingram et al., 2023).

Young people from middle-class and working-class backgrounds who experience social mobility through the expansion of access to higher education undergo fundamental biographical and cultural changes. In many cases, this entails moral contradictions in relation to their social origin and significant dispositional changes (Lehmann, 2014; Ingram and Abrahams, 2015; Reay, 2018). Particularly among those who are the first professionals in their families, the process of mobility is rarely linear or stable. The university experience involves moments of adaptation, modification, and satisfaction, but also conflict, loss, and struggle (Lehmann, 2021). Accordingly, they face a variety of struggles and identity negotiations that lead them to diversify their class dispositions (Abrahams and Ingram, 2013).

In Chile, the situations mentioned above are particularly acute at prestigious or elite universities, which require intensive work on oneself in order to adapt (Álvarez-Valdés, 2023). As a result, the university experience presents itself as an emotional paradox. Anxiety, insecurity, fatigue, and mental health problems indicate a high emotional cost. However, students recognize that they are living a privileged experience that is inaccessible to young people from the same social background (Villalobos et al., 2022).

Canales et al. (2020) propose that, in Chile, these young people represent a new middle class that is far from being “aspirational” and is not exclusively reflected in access to consumption. In many cases, the possibility of entering university is presented to them as an opportunity to “be someone” (Canales Cerón et al., 2016, p. 99). Within this framework, Palma-Amestoy (2025) research shows that higher education is perceived as an external demand, mainly from their families, but also to a large extent from society. In their post-secondary aspirations, they combine an interest in overcoming economic constraints with a high regard for personal effort and merit. From their perspective, a university degree is a way to gain respect and dignity, as it provides access to good salaries, but also to social recognition and the opportunity to “emerge as a person” (p. 11).

The new middle groups have also transformed their symbolic boundaries based on narratives that strongly mobilize the idea of merit and individual effort, driven by a desire to improve (Castillo, 2016). For people who have experienced social mobility, such as first-generation graduates, meritocratic discourse is part of the cultural repertoires they use to make sense of their experiences, mainly through the categories of effort, support, luck, and intelligence (Fercovic, 2022). Furthermore, middle classes have undergone transformations in their identity formations, which has led to tensions between cultural and moral boundaries, especially in relation to their individualisation processes and the work they must do to remain true to themselves and their social origins in contexts of advanced neoliberalism (Méndez, 2008).

Changes in the education system have led to a modification of the class structure, creating new factions within the middle class. These transformations are not only observed at the structural level, but also in terms of educational and occupational aspirations. It is in this context that it becomes relevant to study this particular group, as the literature indicates that these contradictions may be behind the growing feelings of injustice that characterize analyses of middle-class discontent and frustration with the Chilean development model of the last 40 years (Araujo, 2019; Brunner, 2021; Castiglioni, 2021).

## 2 Theoretical framework

### 2.1 Sociology of valuation and evaluation. Conceptual tools for analyzing the moral dimension of social mobility

Sociological analyses of first-generation students have been guided mainly by the concepts of social and cultural capital, and by the dislocations and recompositions of habitus (Beattie, 2018). However, the focus of this research is to take a less-traveled path: the moral interpretation that social agents make of their processes of status passage and class position transformation. Sociology has tended to treat class morality as a subjective perception or a mere convention (Sayer, 2005, 2010). However, I propose an approach based on its most concrete and situated practical expressions through values and evaluation criteria.

As recent professionals, first-in-family graduates construct new ways of assessing reality as they acquire new forms of value that interact with the dispositions learned in their social context. It is on this basis that the question of the formation of valuation criteria becomes relevant, in order to analyze the moral transformations associated with processes of social mobility. Given that self-worth depends largely on how others value us, the unequal distribution of recognition and the legitimacy of our value is associated with class position, which profoundly shapes moral outlooks in terms of ideas, feelings, and judgments about social reality (Sayer, 2010). However, these values never appear in pure form, but rather as practical challenges that place them under strain. Classification in terms of defining or transforming one’s class position, is a process of inscription into moral and cultural value hierarchies, where the sense of self-worth and the worth of others is formed and redefined (Skeggs, 2004). Especially in experiences of social mobility, individuals face a daily struggle for value in that they must constantly demonstrate their own worth in order to be recognised by others (Skeggs and Loveday, 2012).

Dewey (2008) was one of the first thinkers to develop a theory of valuation with significant implications on social theory. In this work, the American pragmatist philosopher argues against the idea that facts and values are ontologically separated, proposing a phenomenological link between knowledge, action, and experience. In his theory, he challenges the idea that expressions of value are mere interjections. Instead, he proposes that values are dynamic processes of meaning attribution and practical orientation that emerge in people’s experiences as practical responses to concrete problems. Thus, valuation is linked to individual desires and interests, but also to collective conflicts and aspirations that are verified and redefined through their implementation. Understood as means adjusted to the effectiveness of results, forms of valuation are not universal entities, but historical, contextual, and plural, where each community and situation produces repertoires of valuation that are tested in action.

Inspired by a number of Dewey’s ideas, the recent development of the Sociology of Valuation and Evaluation (SVE) has reactivated academic attention to the dynamics through which different valuation hierarchies articulate with one another or compete in the categorization of social reality (Lamont, 2012). SVE is not a unitary theory, school, nor a homogeneous group of authors, but rather a broad body of work centered on the question of how individuals structure their frameworks of meaning and relate to values. In an attempt to broaden notions of value beyond the realm of economics and price, this approach examines the practices through which social actors define, categorize, and legitimize the value of individuals or groups in their daily lives (De Munck and Zimmermann, 2014; Cefaï et al., 2015).

Considering valuation as a process, the sociology of economy has taken on a major role. Following the question of how and why value and price are assigned to goods in the market economy, Beckert and Aspers (2011) authored a major work on how moral values are formed and how they interact with economic values. On the other hand, the emblematic works of Zelizer (1978, 1979), and later Fourcade (2007) have shown that the economic experience, both of consumption and of value assignment, is also a process of moral valuation.

Within the sociological analysis of categorization, the work of Desrosières and Thévenot (1988), further developed by Desrosières (2004), on the history of statistics and the construction of social figures was also influential. This research helped to raise awareness of the question of how reality is categorized and the scientific disputes over its stabilization, especially in the classification of phenomena such as unemployment or socio-professional status.

In the field of cultural sociology, the idea of valuation processes also had a significant impact, especially following Bourdieu (1988) work *Distinction*. By studying the criteria and social foundations of taste, he expanded and further developed his theory of reproduction by showing that cultural tastes function as mechanisms for differentiation and the hierarchization of prestige and recognition. Through what he calls cultural capital, social groups engage in practices of symbolic differentiation in the context of disputes over the valuation of cultural goods that allow them to generate or maintain status. The line I take in this work is precisely related to Bourdieu’s influence, which can be considered a post-Bourdieusian approach to the formation of values and judgement criteria.

One of the most productive works in this regard has probably been that of Lamont (1992, 2000). Her research on professional groups from different social classes in the United States and France is consistent with Bourdieu’s proposal, but addresses one of its main blind spots: the moral elements in the orientation of action. Her study on boundary work reveals the existence of different cultural repertoires that legitimize the forms of value used to classify the others. Furthermore, she shows how these forms of value compete for the categorization of reality, drawing on symbolic resources such as strategy, distinctions, and interpretations. Thus, instead of appealing to a universal hierarchy of values, Lamont proposes the existence of competing *heterarchies* in the dynamics of legitimizing values and forms of categorization (Lamont, 2012).

More recently, Heinich (2017, 2020) research provides key insights for a sociological approach to values. Seeking to move beyond the debate on values as intrinsic or extrinsic properties of objects and people, and avoiding the moralistic reductionism of understanding them only as virtues, he adopts a pragmatic approach to the study of valuation acts, including measurement, attachment, and value judgement. Thus, he understands value as the result of the set of operations by which a quality is assigned to an entity. This, however, is not arbitrary. They are shared mental representations, social institutions and relationships that form repertoires. Values are then sustained by the objective qualities and valuation principles of repertoires, which serve as means or tools for giving meaning and value to things, people, actions, and states of the world. Among the different forms of value he proposes, I focus on “value-principle,” specifically on the moral value judgements of what he identifies as the ethics regime.

This theoretical framework attempts to observe inequalities in the form of everyday symbolic struggles, highlighting the role of social processes and power relations that determine the value assigned to individuals, objects, and practices. In doing so, they actively shape inequality by creating hierarchies of worth. In order to distance SVE from the more deterministic uses of Bourdieu’s main concepts, while still addressing his most important questions, it emphasizes the forms of negotiation that social actors mobilize when dealing with specific situations. This does not mean that there are no pre-reflective class dispositions that reproduce structural inequalities. Rather, it highlights people’s ability to move creatively within these constraints, through disputes over valuation and justification in everyday life.

Particularly in the processes of categorization and legitimization of the value assigned to groups or individuals, both Walzer (2001) and Boltanski and Thévenot (1991) account for the simultaneously immanent and transcendent nature of moral judgment, insofar as they always appeal to a greater common good as a form of justification within a situated and contextual injustice. As part of the valuation repertoires, the common good appears as narrative practices of ‘moving to the general’ in which people refer to different spheres of justice to justify their valuations.

Although there are different approaches and schools of thought that focus on forms of valuation, both economic and non-economic, they all share the interest in the ways in which social actors produce meaning and assign moral value. The questions raised by SVE thus revisit classical sociology’s interest in linking morality and social cohesion, but framed as situated and contextual tests. Hence, concepts such as repertoires and valuation are proposed, understood as sets of tools and behaviors that people use in disputes over the recognition and legitimation of value.

This theoretical approach is relevant and pertinent for this research insofar as universities are social institutions that disseminate broader civic and cultural values related to the appreciation of democracy, equality, and meritocracy in a sort of “liberalization” process (Apfeld et al., 2024). Although their social role is under pressure due to changes in the economy and the nature of the labor market, they are still considered one of the main institutions for the transmission of social agendas linked to democracy, economic development, social mobility, and innovation (Pinheiro et al., 2015). From this standpoint, universities can be analysed as ideological apparatuses in the sense that they produce the political socialization of professional roles (Udas and Stagg, 2019). The concept of “public professionalism” proposed by Fleet (2021) for the Chilean context offers important insights in this regard. From his perspective, universities and the specific knowledge of each profession produce a political orientation in professional work, which is then reflected in the professional composition of governments and their ways of attempting to influence the state.

Considering the above, I will mainly use the concepts of value repertoires to study the formation of new values based on university experience, especially in encounters with peers from different social backgrounds. Based on the idea of value as a dynamic process of attributing meaning and guiding action, I observe also the moral meanings that emerge about the profession as a central component of value repertoires. In summary, I will use these conceptual tools to analyse the formation of values as emerging criteria for valuation in processes of social mobility, and value judgements that individuals use to narrate their own experiences of social mobility.

## 3 Materials and methods

This research takes a qualitative approach to answer the question of how first-in-family graduates of public and private universities in Santiago, Chile, develop value criteria and moral hierarchies when evaluating their university and work experiences.

To address this question, two specific objectives were formulated: first, to analyze the value repertoires that emerge in the university experience, specifically regarding the exposure to social diversity; and second, to identify the moral meanings of the degree and professional practice during graduates’ first employment after graduation. In order to achieve this objective, two expressions presented in the theoretical framework will be used: repertoires of values and moral meanings. Following Heinich (2017) approach, I observe mainly the value judgements made by participants and the moral values they use as criteria to narrate their university experiences and entry into the labour market. Furthermore, the analysis of a transition process, or a change in status, also follows this author’s proposal in that it constitutes a “test of value” that students must undergo as they change of status. Regarding the moral meanings of the profession, I use this concept to describe the meanings they produce during the process of evaluating their university and first employment experiences and assigning value both to peers and to their new professional status. A qualitative methodology is particularly relevant for this inquiry, as it focuses on people’s subjective experiences and their social realities through meanings (Flick, 2007, 2015).

The fieldwork took place between July 2024 and November 2025. Twenty semi-structured interviews were conducted, including 11 women and 9 men aged 24 to 30 years. The distinctive feature of this type of interview is precisely its flexibility and focus on the topics outlined in the research objectives. Above all, it seeks a balance between predefined topics and those emerging in the narratives (Flick, 2007), allowing an in-depth study of university and employment experiences. Although the total number of interviews followed the criterion of thematic saturation, the excerpts presented in the results were selected based on their conceptual richness and thematic representativeness. Following the guidelines of Collins et al. (2024) on the definition of cases in qualitative research, I used two main criteria for presenting them: cases that allowed for an in-depth description of conceptually important elements and cases that best illustrate both the repetition of patterns and the significant differences in the participants’ experiences. Thus, the narratives used un the results condense the main moral dimensions that emerged in the interviews, while also defining a common dimension of their experiences and the diversity of their perspectives.

The interviews followed a script of semi-structured questions and covered the entire experience from university to starting the first professional job. I used a script of open-ended questions followed by in-depth prompts to cover topics such as university and career choice, main academic and cultural challenges at university, graduation processes, ways of seeking and adapting to first employment and meanings associated with professional life and activity. In addition, topics related to moral ideas about university education and professional life were addressed. Within this section of the interview, specific questions were asked about what it meant to them to have a university degree and be a good professional. Specific examples from the workplace were requested (e.g., “What does a good professional do?”) as a boundary work exercise to outline the values assigned to these ideas of goodness. Taking a slightly different approach from that proposed by Lamont on boundary work (1992), I am interested in the value criteria used to construct or deconstruct them rather than the boundary itself.

Interviews were conducted in locations chosen by the participants themselves, usually coffee shops near their workplaces or the universities from which they graduated. All interviews lasted between one and a half and two hours. This material was transcribed verbatim and analyzed using thematic analysis (Braun and Clarke, 2012). Specifically, a coding process was carried out using Atlas. Ti 25 to identify patterns of meaning and experiences associated with the aforementioned subjects. This analysis technique can be summarized in four general stages. The first stage is familiarization with the data, during which detailed transcriptions and readings of the material are conducted. Next, significant fragments were coded, combining some predefined codes from the research design with those that emerged from the participants’ narratives. In the third stage, general themes were identified in line with the topics proposed in the interviews. Finally, the codes were grouped within the general themes identified in order to organize and analyze them in detail for presentation in the research report.

As for participant selection, purposive sampling was used (Patton, 2015). The participants were men and women who had graduated from high- and low-social-mobility degree programs, according to the typology proposed by Villalobos et al. (2020). They were also the first professionals in their families and were in their first or second year of employment after graduation. The main reason for working with first-generation graduates is that they are the group that best represents what the literature has called the new middle classes. Moreover, as they are the first professionals in their families, the changes and disruptions with their social origins are more significant, as is the identification of their experience as a form of relative social mobility, especially in relation to their parents’ educational and occupational trajectories and the family’s overall income level. It is important to mention that, given the scale and methodological characteristics of this research, it is not intended to produce generalisations, but rather an in-depth study of a specific case of social mobility, that of first-generation university students in Santiago, Chile.

The selected degrees were medicine, law, and business administration for the high-mobility typology; and psychology and sociology for the low-mobility typology. The objective of selecting high- and low-mobility degrees was to observe whether there were differences or similarities. In addition, psychology is one of the degrees with the highest enrollment rates nationwide, making it relevant for observing an “average” job insertion process compared with high-mobility degrees. It is important to note that no significant differences were found by type of university or degree program. Some more pronounced elements were observed according to gender and type of institution, which are described in the results.

Following the same typology mentioned above, the study focused on new private universities, referring to those established in the 1990s and early 2000s, and traditional state and private universities in the metropolitan region, which are also considered elite institutions. The higher education system in Chile is organized around three types of institutions: universities, professional institutes, and technical training centers. It is, however, characterized by significant segregation based on students’ socioeconomic status (Garcés et al., 2024), with universities concentrating those with greater resources. So-called state universities, in turn, were established as public law corporations, yet all higher education institutions in Chile charge tuition and monthly fees. Therefore, the term traditional can refer to either state or private universities, which does not imply free or universal access. Several financial support systems have been created in recent decades, either in the form of debt or scholarships (Santelices M. et al., 2019). For this study, I focused particularly on beneficiaries of the free tuition law, in place since 2016, which targets the 60% most vulnerable in the country according to the social protection scoring system used by the Ministry of Social Development. The selection of institutions covered by the free tuition law also ensures socially diverse university environments. This is essential for investigating university experiences that involve encounters with peers from different social backgrounds and greater contrast with the dispositions and habits acquired in one’s own social background (Table 1).

**Table 1:** Sample characterization.

**Participants**	**University type**	**Degree**
Interviewee 1	New private	Psychology
Interviewee 2	New private	Psychology
Interviewee 3	New private	Psychology
Interviewee 4	New private	Psychology
Interviewee 5	New private	Law
Interviewee 6	New private	Medicine
Interviewee 7	New private	Psychology
Interviewee 8	New private	Law
Interviewee 9	New private	Law
Interviewee 10	New private	Commercial engineering
Interviewee 11	New private	Commercial engineering
Interviewee 12	Private traditional	Law
Interviewee 13	Private traditional	Law
Interviewee 14	State traditional	Law
Interviewee 15	State traditional	Commercial engineering
Interviewee 16	State traditional	Law
Interviewee 17	State traditional	Medicine
Interviewee 18	State traditional	Sociology
Interviewee 19	State traditional	Commercial engineering
Interviewee 20	State traditional	Commercial engineering

Source: Own elaboration.

In terms of ethical protocols, the project was approved by the ethics committee of the affiliated university. In addition, all interviews included written consent forms explaining the objectives of the research and the possible risks associated with recalling or reflecting on sensitive moments. Given this potential scenario, participants could withdraw from the study at any time without having to justify their decision or face any consequences. Finally, all participants’ names were replaced with fictional ones, and all proper names of cities, institutions, and neighborhoods were anonymized.

## 4 Results

### 4.1 The experience of inequality and value formation at university. From initial shock to expanded horizons

Although universities are institutions that teach values through the curriculum, educational projects, and the hidden curriculum, in this section I address how new values are generated in the university experience of encountering peers from different social backgrounds. In particular, in the case of first-in-family students, these values interact with those socialized in their social contexts, producing a strained openness and expansion of their repertoires in different dimensions, ranging from aesthetic tastes to a sense of justice and the valuation of diversity. In this way, university creates new outlooks, which they use to evaluate their experiences, their friendships, and themselves as individuals.

A primary empirical observation is that encountering social diversity in higher education fosters value repertoires tied to social heterogeneity. Such inequalities, predominantly socioeconomic, are engaged with both critically and virtuously. On the one hand, it broadens the appreciation for diversity and the ways in which others are valued, serving as a form of personal enrichment. On the other hand, it produces an awareness of the inequality that characterizes their original social contexts. Alongside this notion of inequality, ideas of injustice and critiques of structural inequality emerge.

Social diversity is experienced as a cultural shock that reinforces certain ideas of injustice and inequality, but also broadens and expands ideas about oneself and others. Participants generally reported a process of *symbolic disarmament*, in the sense that they moved from more rigid, generalized perceptions of people from different social backgrounds, especially those they identified as affluent or privileged, to more nuanced assessments based on concrete experiences of encounter and friendship.

Gloria’s narrative is very illustrative in this regard. Her experience studying law at a traditional state university created tensions with the perceptions she had about upper-class people. Being able to interact with students from privileged backgrounds was a real shock to her, especially those whose privileges were inherited, as reflected in her reference to “fancy last names.”

The change was extremely difficult for me, particularly because I was very prejudiced against people with more resources. I had a strong dislike for fancy last names. I’m in a major that, in a way, is quite elitist, bringing together people who come from families of lawyers. So, it was quite a significant shock.

In her account, her university experience and encounters with those she considered privileged allowed for a kind of transformation of her initial ideas through a valuational openness. At one point in the interview, Gloria recalls how she met the person she now considers her best friend. During final exams, they were the only two students who attended an elective class, which ultimately never took place because the professor cancelled the session without notice. Finding themselves alone in the classroom, Gloria “had no choice but to talk to her”. In that casual exchange, she realized that her classmate was not only very kind and friendly, but also “reasonable” and “intelligent” in emotional terms. A significant point here is the use of moral and emotional categories to define the value of others. Gloria does not value her friend in terms of status or academic performance, but rather for her relational and interaction skills, producing a *moral reconfiguration of the other*.

We became very good friends, and then I found out that she came from a family of lawyers who live in [name of a wealthy neighborhood in eastern Santiago] with an important surname, and I thought, “She's fantastic [laughs]; she's an incredible person, really reasonable, very intelligent; not the kind of intelligence that gets good grades, but a very high emotional intelligence”.

The account of Lucas, a commercial engineering graduate from a traditional state university, is equally insightful. “What happened to me when I entered university was that my world opened up,” he states after a brief silence in which he reflects and tries to sum up in a single sentence what university meant to him. The idea of “openness” reappears to explain the effect of meeting people from different social backgrounds. Remembering his first year at university, he remarks: “there are so many different realities, people who are very different from you, people from different socioeconomic backgrounds, seeing that the world is so diverse, it’s shocking.” For him, diversity is both enriching and shocking. He elaborates more on this when recalling the first time he visited the house of one of his classmates from the wealthiest neighborhood in the capital. During his visit, he was not only impressed by the materiality and spaces, but also by the distance that exists between the lifestyles of people who do not have that wealth. Directly observing this privileged reality makes him more aware of injustice, which also facilitates a critical judgment.

When you go to people's homes, you realize that they’re very different. It's shocking, but it's also nice. I think that, at that point, I opened up, gave myself the chance to learn and, deep down, you mature. I understand that initial hatred, and I respect it in a way…. I really don't care where I live, but you realize that not everyone has the same options and that it's not their fault.

Lucas’s narrative shows that this openness to difference also allows for the development of a sense of inequality, recognizing and criticizing the country’s structural inequalities. Of particular relevance in this context is that his critique is not directed personally at his privileged classmate, but rather at broader injustices for which people are “not to blame.” Lucas’s critique is based on a general idea of injustice in relation to society as a whole, specifically in reference to the sphere of economic justice. Through a process of “moving to the general” (Boltanski and Thévenot, 1991), he shifts from a specific experience to a broader concept of justice that allows him to justify his discontent. This critique is also sustained by feelings of anger that are later tempered through the disposition to learn about these new contexts.

The value of personal effort emerges as a core value in defining both oneself and others. Gloria, a lawyer who attended a traditional state university, illustrates this clearly in her story. For her, effort is a personal quality, a way of navigating university, but also a way of valuing being there. Likewise, the value of personal effort becomes a criterion for distancing oneself from those who feel it is natural to go to university.

There is a type of student at university who doesn't value studying. For them, it's perfectly normal to go to university, enjoy university life, and that's it. That shocks me precisely because it took me a lot of effort to get here and I really value being here…. I've always had to work really hard …. I like to say that I'm not a brilliant person, but I am a hard-working person.

Here, the idea of meritocracy is not linked to deserving something, but rather to a way of being and behaving at university, a disposition toward university culture that demonstrates valuation through effort.

Finally, notable shifts are evident in aesthetic standards and clothing choices. An appreciation for carefully selected outfits, or combinations that convey “meaning” in one’s appearance, emerges as a learned value. Although the emergence of new aesthetic criteria is shaped by the cultural codes of the university, participants perceive it largely as an extension of their own sense of taste, or what they regard as “nice” and “comfortable,” as a new way of valuing self-presentation. This is particularly evident in the story of Gloria, for whom developing these new criteria was a learning experience.

I feel like I didn't know how to dress before [laughs]. Especially in this faculty, people dress very well [laughs]. You can tell you thought about it when you put it on. It makes sense visually…. I arrived wearing my jeans, my sweatshirt, and my Converse, and I said, “Wow! This is weird,” and I changed the way I dressed a little because I thought the other way was really nice, not because I felt like I didn't fit in.

Rocío, a psychologist from a private university, shows a more gradual development. Although she acknowledges the “standard” of the context, how her female classmates dressed for university, the development of her taste for makeup and clothing stems from a desire to try something new, until it becomes something enjoyable, like a daily routine activity centered on fun.

At university, girls worry about looking good. And I would get up, wash my face, take a shower, and that was it. Then I said, “Let's try it for a while, and if I don't feel comfortable, I'll go back to how I was.” And then, you know what? I liked it! The routine of choosing the right clothes, putting them together, curling my eyelashes, putting on lipstick. That started to change, but it's not like I lost my identity or anything.

Rocío categorically separates her new aesthetic tastes from her identity. For her, this did not mean a transformation of herself as a person, but rather the adoption of a new habit linked to aesthetic pleasure and working on herself. As in Gloria’s experience, there is a strong element of context, how others dress, and a certain risk of transforming oneself into something one is not. However, what is valued is the incorporation of these new criteria as a way of participating in the collective dynamic of “authenticity” (Méndez, 2008) doing something unique and enjoyable.

The results of this section highlight the culture shock experienced by first-generation students at university, which has several effects. First, they become more aware of the gaps in which they grew up, especially material and educational gaps, which leads to the development of critiques of the country’s structural inequalities. Second, graduates experience an expansion of their repertoire of values and understanding of themselves and others as a result of their exposure to diverse social environments. Thus, students move from feelings of inadequacy and discomfort to an appreciation of difference and diversity. Although these results were observed in graduates from all institutions, they are more pronounced in traditional elite universities. Finally, rather than passive reproduction of inequality, this result illustrates Dewey’s notion of valuation (2008) as a dynamic adjustment to concrete challenges, where recognition and legitimacy are unevenly distributed but actively contested.

### 4.2 Recognition, self-worth and economic stability. Conflicting values about social mobility

Universities do not only transmit so-called liberal values. Through professional socialization, they transmit moral ideas about how to perform a profession and its potential contribution to society. In this section, I argue the possible connections and differences between moral values derived from social class, mainly through the family, and those developed within the university professional training. It is, in short, the question of what they considered to be the function and purpose of a university degree.

Considering the perspectives of the research participants on their parents, the first thing that can be observed in the narratives is the dual function that families assign to university: one linked to material life and economic stability, and the other related to status. A key finding of this section shows that the expectation of university as a tool for improving quality of life comes mainly from families. However, they also stress the importance of higher education as a tool to gain recognition and create self-worth.

Rocío, a psychologist who graduated from a private university, describes how her parents conveyed the importance of studying. They referred to how difficult it is to find a job and the opportunities that a profession opens up in terms of job satisfaction. Her family also emphasized the prestige that a university degree brings. A noteworthy aspect of this case is that social recognition is related to the possibility of receiving good treatment, along with material stability. Thus, recognition reflects a value of oneself as worthy of dignified treatment.

They know that without a degree it’s difficult to find a job and they struggle to feel that satisfaction. So, for them, university gives you prestige, it gives you fair treatment, and it gives you stability…. You can make a living and be financially independent.

In her experience, there is a transmission of a meaning associated with financial autonomy. However, autonomy is not presented in terms of expanding individual possibilities, but rather as a way of avoiding dependence, especially that which may arise with a partner. Rocío illustrates this with a narrative about her mother, thus highlighting the transmission of gendered meanings regarding autonomy.

My mom always tells me never to depend on any man, even though she's never had any problems with that, but she has friends who have, so she always says that to me.

For Fernanda, a psychology graduate from a private university, the role of conveying the importance of higher education is directly associated with her mother. The duality of economic stability and personal fulfillment also appears, but in relation to the possibility of enjoying one’s professional activity. She suggests that this is only partly true, since work always involves fatigue and stress. Her view, unlike her mother’s, is that this burden becomes more tolerable when you enjoy what you do.

My mom used to say, “you’ve got to study, because if you do, you’ll make good money and people will treat you better.” I think that’s partly true. You really do things better when you like them, but you still work, you still get tired, but I feel like it hurts less if you do what you love.

The university degree as a means of gaining respect and good treatment also appears in her testimony. These findings indicate that they represent a demand for dignity and a tool that might produce it. The degree thus becomes a way of acquiring this value regardless of one’s origin and background.

As an economist who graduated from a traditional state university, Cristian argues that status and the possibility of a quieter life were always the meanings his parents passed on to him. University as a minimum foundation that will provide future economic security, where you can access “important people,” develop skills, and acquire status, are the elements that will ultimately lead to an easier and more secure life.

My parents always told me that university was the bare minimum, that I had to make the most of my abilities, meet important people; they had this very status-oriented view of university. The other thing is the economic issue. They always told me, “Life is hard for everyone, and going to university will give you a minimum foundation.”

The families of the participants did not always view university positively or uniformly among both parents. In the experience of Isidora, a lawyer graduated from a private university, her mother never agreed with her studying a traditional degree such as law. ‘My mother says it was too much sacrifice for a very traditional degree’ stated. Instead, her expectations were for her daughter to complete a short degree, learn English and travel the world. In contrast, her father dreams that one day his daughter will become a public prosecutor. However, when she graduated and began looking for employment, the importance that both parents placed on her professional degree was related to income level and savings capacity. On the contrary, and without dismissing the importance of income, Isidora’s expectation is to become independent from her parents and work in a job she enjoys.

What they want most is for me to start working so I can begin saving. But I don't want that; I want to work so I can become independent.

And what do they want you to save for?

To buy something, a house or a flat. To be honest, I'm not really interested in owning property at the moment. I want to pursue a career in something I enjoy.

Natalia’s narrative also shows the importance her parents place on university degrees, especially as a medical graduate from a private university. She is an only child, lives with her mother, aunt and cousin, and devotes practically 100% of her day to studying. At the time of the interview, she was working as an intern at a public hospital and pursuing a medical specialisation. However, she has been exclusively dedicated to her studies since her undergraduate days. In her narrative, she claims that her mother always highlights the high prestige of the university degree as well as her concern about the lifestyle that such a demanding career can entail, with high levels of stress and the requirement for continuous training, which can lead to a significant sacrifice in terms of well-being.

At some point, [my mother] would tell me that I was pushing myself too hard, that I should try to rest, and I would say, ‘I can't’ (laughs)…. I always have a presentation to give on such-and-such a day, I have to study for the test, I have to do this and that, it never ends. So, on the one hand, for her, university and getting a degree, becoming a doctor, was the ultimate goal. But on the other hand, perhaps it affects my well-being a little.

Both Isidora’s and Natalia’s stories show that their families convey about professional life highlight the instrumental nature of the degree, mixed with concerns about the lifestyle that comes with the career they studied. Thus, in both cases, alongside the value placed on improving material life and gaining prestige, there are also hierarchical ideas about desirable lifestyles based on the level of well-being they allow.

Security, status, well-being and the possibility of self-fulfillment are some of the values transmitted in families regarding the function of university education. It is worth emphasizing that a professional degree is never related solely to its potential return, but rather to the type of life it allows one to build and the self-worth it generates. From the perspective of the interviewees, what families categorize as status is the possibility of being “important” in terms of deserving of good treatment and secure living conditions. As general findings from this section, we see that the degree is not only a credential but a moral symbol deeply embedded in expectations of self-fulfillment. Thus, university education is both a tool for generating income and a means of personal fulfillment through the potential development of a vocation.

### 4.3 Moral meanings of professional activity and the formation of social consciousness about the common good

In contrast to the moral values that come from families, students develop their own as they go through university and begin to work. The social role of the profession was a cross-cutting theme in the interviews, both among graduates of state and private institutions. Without neglecting their interest in salary and stable contracts, when asked what professional activity meant to them, they all expressed a clear interest in its social purpose. This was mainly related to their own experiences of precariousness and inequality. Thus, social interest was, in fact, a sense of having to give something back to their communities.

Although this social orientation is sometimes presented as something that they “always had,” when analysed in detail it can be seen that it is built on specific experiences of direct contact with the beneficiaries of the profession. Gloria, a lawyer from a traditional state university, recounts her experience working with a community in south-central Chile after a major wildfire that left many people homeless. As part of a practical course, she worked to help process property titles with those affected so that they could apply for state aid to rebuild their homes. She considers this experience transformative in terms of her criteria for defining the meaning of work.

That fieldwork changed my life…. I like being able to help people, work with people, talk to people. I believe I have to be good at what I do, because if I'm good, I'll be able to help more people. That's my vision now. I like helping people; I think that's what fulfills me the most. I don't need to be a congresswoman; I don't need that kind of recognition.

That experience triggers a social vocation that contrasts with status in the sense of being “important” and “recognized.” In other words, social meaning serves as a differential value aimed at “imbuing” professional activity with a meaning that is considered to be of higher value. Furthermore, this social sense guides the idea of being good at what you do.

Through these moral meanings, they do not seek only to do good, as it is not merely a matter of moral motivation. Here, different dimensions of what is considered meaningful professional activity interact. On the one hand, there is a broad sense of employment that goes beyond the individual and, on the other hand, there is a sense of self-realization that is oriented precisely toward individual well-being. Moral meanings are therefore related to a way of understanding professional practice rather than the discipline in the abstract, and generate a hierarchy of values that allows for the evaluation of one’s own performance and that of others.

Cristian’s story is also indicative of this trend. He is a commercial engineering graduate from a traditional state university and works in a support center for low-income students. In the student affairs department of a university, he works directly with them, providing academic support and guidance. In his interview, he articulates a social vision of what he calls a “professional project” in contrast to a career driven exclusively by profit.

Money is important, and I want to live comfortably, but my professional project goes much further than that. I want to do things with social meaning, things that make a contribution. That's why I work here at the university. It's not the best job for a commercial engineer, but I like what I do because I provide academic support, I work with students, and I really enjoy interacting with them.

As with Gloria, Cristian highlights the value of proximity. He emphasizes the richness that comes from being able to talk to the student directly and see the concrete impact of his efforts. This also connects with the idea of the profession as a tool for public advocacy and for “contributing to society”. However, this is not solely vocational or purely altruistic. On the contrary, Cristian mentions that this meaning is valued on the basis that the degree is a certainty, a “minimum floor” that allows one to “get by” and “live” in case things do not turn out as planned.

The aforementioned sense of duty leads to different forms of practical engagement, ranging from becoming the doctor for the extended family and closest neighbors, prioritizing work in public hospitals, to providing legal aid or psychological assistance to those unable to afford it. These forms of professional practice produce what I identify as *professional activism*. Based on the notion of public professional proposed by Fleet (2021), I propose that professional activity as political action is not only oriented toward work in the state, but also toward more direct forms of territorial involvement.

Professional activism generally takes place outside formal working hours, as volunteer work in the communities where they grew up. Lucas, a commercial engineer from a traditional public university, currently works as a data analyst in a state ministry. In his time outside work, he, together with a group of friends who were also able to attend university thanks to the free tuition law, decided to set up a pre-university program in their neighborhood of origin. There, they train high school students to take the Higher Education Admissions Test, offering free classes in math and language.

I volunteer at a pre-university program in [name of neighborhood] together with other people. We set up a free social pre-university program and we work there…. The cool thing is that it's people from there, who are professionals and who have experienced a series of obstacles, which makes them want to help other people who come from the same background.

Lucas mentions that they do not receive financial assistance. They secured a small room in the municipality that already had the minimum equipment to hold classes. In many cases, they cover the cost of materials and obtain practice tests. At the time of the interview, they were trying to establish a legal foundation so that they could apply for funding as an institution.

Mauricio’s narrative also shows a form of professional activism, but one that is more influenced by the territory and region where he was born. After graduating with a law degree from a traditional private university, he decided to return to his hometown in southern Chile. At first, he provided independent legal advice in real estate. After a few months, he felt that this was insufficient because and wanted to contribute more broadly. He, together with a group of local friends, then decided to open a non-profit foundation to promote access to culture, especially reading and artistic works. At the time of the interview, Mauricio was the executive director of the organization. From there, they had managed to secure some funding to set up a community library and a free co-working space. He combines this activity with his legal consulting work, which remains his main source of income.

I’m the co-founder of a foundation that aims to promote access to culture in isolated areas…. I have a strong vocation for social work, especially in the environment where I grew up. [Name of his town in southern Chile] is a town that never changes. Realizing that there’s so much you can do with people in areas outside Santiago motivates me.

Based on these narratives, we see that professional activism is not only about the idea of transformation and its contribution to personal fulfillment through the social orientation of professional activity.

This section shows that, for the group of first-generation graduates with whom I worked in this study, the professional activity at this point in their careers and biographies involves an idea of justice, of leveling the playing field by directly supporting others with professional knowledge applied freely and voluntarily. This, of course, is challenged by the difficulty of combining formal employment and volunteer activity, especially when participants begin to have greater financial autonomy and higher expenses associated with leaving their parents’ home.

## 5 Discussion

The analysis of first-in-family graduates’ trajectories from university to employment highlights the complex interplay between structural inequalities, individual agency, and morality. By examining both the formative experiences at university and the moral meanings attached to professional activity, this research situates social mobility not only as an economic or occupational process but also as a moral and relational transformation.

In terms of value formation at university, the results show that students’ initial encounters are marked by insecurity and a sense of inadequacy. Yet these experiences are not static. Over time, graduates start valuing diversity, alongside personal effort and new aesthetic tastes, transforming initial shock into expanded horizons of values.

This is precisely what Apfeld et al. (2024) propose as the liberalizing effect, especially in relation to the appreciation of difference and broadening of outlooks through exposure to social diversity. New forms of value that emerge in the university are always somewhat nuanced, in the sense that they are not pure forms of value, but rather criteria that facilitate the symbolic modulation and navigation of the university experience and entry into employment. What we see, then, is a process of moral and valuative openness toward oneself and others. The appreciation of diversity is, however, coupled with a critique of economic injustice (Walzer, 2001) and the development of what Bottero (2019) calls “sense of inequality”.

Although these value repertoires emerge in a situated and contextual manner, the possibility of critique and appropriation accounts for broader ideas of justice and communality (Boltanski and Thévenot, 1991), in terms of enabling participation in the common good, either by incorporating new values or by criticizing injustices that become more visible in the context of cultural contrast. In this sense, the expansion of horizons is always accompanied by the recognition of limits, a tension that defines the moral dimension of social mobility. This relates to Morton (2019) work, which is probably one of the main references in the field of morality and social mobility. Specifically, his research with young people in the United States shows that there is an ethical cost to upward social mobility processes, which mainly affects their family relationships, their sense of cultural belonging, identities and, above all, their worldviews, ways of valuing, and meanings of their life experiences.

Furthermore, the results indicate that the transition to professional life for these first-generation graduates and the possibility of building a career represents an opportunity to demand and acquire dignity and respect. Considering Sennett (2003) seminal work on this subject, the desire for respect as a form of moral value grows in contexts of high inequality. This is further complemented by the consideration of a university degree as a means of generating self-worth based on social recognition, which is expressed primarily in the expectation of fair treatment. Honneth (1997) definition of recognition is undoubtedly important for understanding these results. Based on his definition of love, legal relations and social esteem as the three fundamental spheres of mutual recognition, these findings show that the value repertoires and moral meanings of the young people who participated in the study are relate mainly with the struggle for social esteem, insofar as they appeal to a horizon of shared values within which both qualities and achievements of oneself and others can be evaluated. I therefore propose that these disputes guide the management of the transition to employment, outline a possible career path and allows them to give meaning to their experiences of social mobility.

The moral meanings of the degree also reflect an interaction between values socialized within the family, mainly vocational and material, and those socialized during university and early professional experiences, such as the social contribution. We then see the emergence of new dispositions associated with a process of professional socialization that enriches and challenges the initial dispositions. In the experience of these first-generation graduates, professional socialization at university acts as a process of individual recomposition (Darmon, 2016).

It is noteworthy that, although all professions convey and socialize an ethic of service, technical foundations, and a jurisdiction that gives them a certain degree of autonomy (Schinkel and Noordegraaf, 2011; Abbott, 2003), the participants from all degree programs and universities share a sense of social responsibility and orientation toward others in their professional activity. Furthermore, a sense of duty and personal fulfillment from helping others were cross-cutting themes in the interviews.

## 6 Conclusion

This research shows that first-generation graduates that participated in this study have access to, and internalize, new forms of valuation as they pass through socially diverse universities and begin their first employment experiences. These values become criteria for judging others, their profession, and their jobs. Furthermore, they extend these values to ways of understanding the profession, defining job meanings associated mainly with their social role and public contribution. This, in turn, can lead to professional activism practices, as a concrete form of professional engagement in their neighborhoods of origin. In this context, Bourdieu’s class habitus is visible in its transformation and change, along with the incorporation of new forms of value. Consequently, their experiences of social mobility are not only about the development of cultural preferences, but also about political and moral positions in the construction of their worldviews (De Keere, 2020).

Taken together, the findings suggest that social mobility through higher education is experienced as both an expansion and a burden. For these first-in-family graduates, university enables new valuative repertoires, expanded horizons, and the possibility of reinterpreting one’s social position.

In broader terms, the research underscores that social mobility cannot be reduced to occupational outcomes or income. It involves moral transformations and redefinitions of self-worth. Specifically, university-to-employment transition is a process of moral positioning: graduates seek not only to improve their material conditions but also to achieve recognition. Consequently, the promise of higher education is about economic advancement, but also about recognition, justice, and personal fulfillment.

Finally, the study contributes to the sociology of valuation and evaluation by showing how moral categories are mobilized in everyday practices of meaning-making. These categories are not abstract but situated, emerging from the lived experiences of students negotiating recognition and self-worth in stratified educational institutions. This research invites further reflection on how educational systems shape not just careers but moral worlds and social imaginaries of possible futures.

## Data Availability

The original contributions presented in the study are included in the article/supplementary material, further inquiries can be directed to the corresponding author.
